# Spatio-Temporal Pattern Analysis of African Swine Fever Spreading in Northwestern Italy—The Role of Habitat Interfaces

**DOI:** 10.3390/ani15192886

**Published:** 2025-10-02

**Authors:** Samuele De Petris, Tommaso Orusa, Annalisa Viani, Francesco Feliziani, Marco Sordilli, Sabatino Troisi, Simona Zoppi, Marco Ragionieri, Riccardo Orusa, Enrico Borgogno-Mondino

**Affiliations:** 1 Department of Agricultural, Forest and Food Sciences (DISAFA)—GEO4Agri DISAFA Laboratory, University of Turin, 10095 Grugliasco, Italy; samuele.depetris@unito.it (S.D.P.); enrico.borgogno@unito.it (E.B.-M.); 2 INVA Spa, 11020 Brissogne, Italy; 3 Azienda USL della Valle d’Aosta, S.C. Sanità Animale, 11020 Quart, Italy; aviani@ausl.vda.it (A.V.); mragionieri@ausl.vda.it (M.R.); 4 Istituto Zooprofilattico Sperimentale Umbria e Marche “Togo Rosati” NRL for Swine Fevers, 06126 Perugia, Italy; f.feliziani@izsum.it; 5 Ministero della Salute, 00144 Roma, Italy; m.sordilli@sanita.it; 6 Istituto di Gestione della Fauna APS, 80126 Napoli, Italy; sabatinotroisi@gmail.com; 7 Istituto Zooprofilattico Sperimentale del Piemonte, Liguria e Valle d’Aosta (IZS PLV) S.C. Diagnostica Generale, 10154 Torino, Italy; simona.zoppi@izsplv.it; 8 Istituto Zooprofilattico Sperimentale del Piemonte, Liguria e Valle d’Aosta (IZS PLV) S.C Valle d’Aosta—CeRMAS (National Reference Center for Wildlife Diseases), 11020 Quart, Italy; riccardo.orusa@izsplv.it

**Keywords:** African Swine Fever (ASF), GIS, geostatistics, spatio-temporal modeling, habitat interfaces, land cover, risk assessment, wildlife, farms, Piedmont and Liguria (NW Italy)

## Abstract

**Simple Summary:**

African swine fever (ASF) is a highly contagious viral disease affecting domestic pigs and wild boars, causing severe economic and social impacts due to high mortality and the absence of a vaccine. This study uses geospatial technologies, particularly GIS-based spatial mapping, to monitor and analyze ASF outbreaks in northwestern Italy (Piedmont and Liguria). By considering factors such as pig density, proximity to wildlife, and environmental conditions, high-risk areas and disease spread patterns were identified. Results show that central–western areas exhibit higher ASF risk, associated with dense wild boar populations and favorable environmental conditions, while outbreak emergence is delayed in peripheral regions. These findings highlight the importance of local environmental variables in shaping disease dynamics. The study demonstrates that geospatial modeling can guide targeted surveillance, prevention, and resource allocation strategies, supporting more effective disease management. Incorporating these approaches into ASF control programs can improve early detection and inform decision-making. Future research could enhance predictive models by integrating additional datasets, further supporting their applicability across different regions.

**Abstract:**

African swine fever (ASF) is a highly contagious viral disease with significant impacts on domestic pigs and wild boar populations. This study applies GIS-based spatial analysis to monitor ASF outbreaks in northwestern Italy (Piedmont and Liguria) and identify areas at increased risk. Key factors considered include pig density, wildlife proximity, and environmental conditions. The spatial analysis revealed that central–western municipalities exhibited higher risk due to favorable environmental conditions and dense wild boar populations, while peripheral areas showed a temporal delay in outbreak emergence. Mapping the spreading rate and habitat interfaces allowed the development of a spatial risk model, which was further analyzed using geostatistical techniques to understand disease dynamics. The results demonstrate the effectiveness of geospatial modeling in identifying high-risk zones, characterizing spatio-temporal patterns, and supporting targeted prevention and surveillance strategies. These findings provide actionable insights for ASF management and resource allocation. Future studies may refine these models by integrating additional datasets and environmental variables, enhancing predictive capacity and applicability across different regions.

## 1. Introduction

African swine fever (ASF) is a double-stranded DNA virus belonging to the family *Asfarviridae* and a highly contagious and often fatal viral disease that affects domestic pigs and wild boars [1,2,3]. Moreover, it poses a significant threat to the global pig industry, with severe economic and social consequences [4,5,6,7]. At present, there is no available vaccine, and the mortality rate among affected animals can reach up to 100% [8,9,10]. The virus is known for its strong environmental resilience and high transmissibility [11,12]. ASF has been endemic in sub-Saharan Africa since its initial discovery [1,3,13]. In Europe, the disease was historically confined to the Italian island of Sardinia (1995–2007) [14,15,16]. However, a 2007 outbreak in Georgia triggered a significant spread across Eastern Europe, impacting both domestic pigs and wild boars in neighboring countries [13,17]. The disease entered the European Union in 2014, initially affecting wild boar populations in the Baltic states and Poland. Subsequent years witnessed a continued spread within the EU and neighboring non-EU countries [12,18]. More recently, ASF outbreaks have emerged in Asia, Oceania, and several American countries [19,20,21,22,23]. Outbreaks of ASF can also trigger strict trade restrictions, severely impacting the export of pork products and disrupting global supply chains. This can lead to financial losses for pig farmers and the entire pork industry [4,23,24,25]. Strict biosecurity measures on farms are required to prevent the introduction and spread of ASF [26,27,28,29]. These measures include (a) movement controls: limiting the movement of people, vehicles, and animals onto and off the farm; (b) hygiene and sanitation: implementing rigorous cleaning and disinfection protocols; (c) feed and water security: ensuring the safety and integrity of feed and water sources; and (d) surveillance and early detection: promptly implementing robust surveillance programs to detect and report any suspected cases of ASF [30].

While ASF does not directly infect humans, it can indirectly impact public health through food shortages [22,31,32]. Outbreaks can disrupt pork production, potentially leading to shortages and price increases in pork products [4,33]. Additionally, ASF outbreaks can also have significant socio-economic consequences, including job losses, poverty, and food insecurity [5]. Collaboration between governments, the pig industry, and researchers is vital for controlling and preventing the spread of ASF [11,19,34,35].

In Italy, ASF has been present in Sardinia since 1978, affecting both farmed pigs and wild boar populations, but it has remained confined to the island [14]. Recently, efforts to combat the disease have been significantly improved, resulting in a notable positive shift in the epidemiological situation [36]. In October 2023, restricted zone II was lifted, confirming the absence of viral circulation among wild boar populations [14]. Specifically, in Italy, Restricted Zone II, as defined by EU regulations, specifically refers to areas where ASF is present only in wild boar populations. It is worth noting that an important source to distribute the disease is represented by guest workers who can accidentally become direct or indirect carriers of the virus through trampling and abandoning contaminated food products of porcine origin. This means that while ASF is circulating among wild boars, there are no confirmed outbreaks in domestic pigs within these zones. The implementation of these zones is crucial for controlling the spread of ASF and minimizing its impact on both wild and farmed pig populations. By September 2024, Sardinia was officially declared free of ASF [12].

Recent outbreaks of ASF in northwestern Italy, particularly between the regions of Piedmont and Liguria, highlight the complex interplay of local factors contributing to disease emergence and spread. The initial outbreak in Ovada, a town characterized by a dense network of truck rest areas and proximity to major transport corridors leading to the Port of Genoa, suggests a strong link between ASF introduction and anthropogenic activities. The presence of discarded pork products, including potentially contaminated swine-derived sausages, by truck drivers and holiday travelers along highways and forested areas may have facilitated indirect transmission to wild boar populations. These animals, which often forage near human settlements and agricultural fields, are highly susceptible to ASF and serve as effective reservoirs. Additionally, the region’s landscape features dense woodlands with frequent interfaces between natural habitats, farmland, and urbanized zones, further complicating containment efforts. The limited understanding of wild boar population density and movement patterns exacerbates the challenge, underscoring the importance of targeted surveillance and biosecurity measures. These localized dependencies, both infrastructural and ecological, have played a critical role in the emergence and propagation of ASF in this transregional corridor. Similar patterns of strong local dependence have been observed in other countries affected by ASF, where regional transport networks, waste management practices, and habitat interfaces contribute significantly to disease dynamics and pose challenges to effective control strategies [37].

The first confirmed case outside Sardinia was reported on 7 January 2022, by the National Reference Center for Swine Pests (CEREP) at the Istituto Zooprofilattico Sperimentale in Umbria and Marche [38]. A wild boar carcass found in the municipality of Ovada, in Alessandria Province (Piedmont), tested positive for ASF. Shortly afterward, the virus was detected in several wild boar carcasses in Liguria, specifically in the province of Genoa. On 5 May 2022, ASF was detected in Lazio, in a dying wild boar found in a natural reserve in the northern part of the Rome metropolitan area, along with other carcasses in its proximity. A suspected ASF case was reported on May 26 in an injured wild boar in the province of Rieti, though after over five months of surveillance, viral circulation was ruled out. On June 9, the disease was also found in a semi-wild pig farm in Rome, located near the area where the first infected wild boar carcass had been discovered. Over a year later, in May 2023, ASF was confirmed in wild boars in Reggio di Calabria, and a few days later, two semi-wild pig farms in Africo, Reggio Calabria, were also affected. That same month, ASF was detected in wild boar carcasses in Campania. In June 2023, ASF was identified in wild boars in Lombardy, in the province of Pavia, and by the end of August, the disease was confirmed in several pig herds in the same region. In September 2023, three cases of ASF were confirmed in the province of Nuoro, attributed to genotype II, which is responsible for the current epidemic in mainland Italy and Europe, but had never been detected in Sardinia. Following the confirmation of ASF in both pigs and wild boar, infected zones for wild boar, along with protection and surveillance zones, were promptly established [12].

In particular, the movement of guest workers both within national borders such as those traveling between Southern and Northern Italy, and from Eastern European countries has played a crucial role in shaping patterns of exposure and transmission. Many of these individuals are employed in the senior care sector, which represents a high-risk setting due to the vulnerability of the population and close, prolonged contact. Notably, a significant portion of these workers bring food from home, which may contribute to the unintentional spread of pathogens, especially when combined with suboptimal sanitary conditions or informal employment arrangements. These socio-economic and behavioral factors underline the importance of considering human mobility and everyday practices in epidemiological models and public health planning [39].

Furthermore, lack of centralized political coordination and the fragmentation of administrative responsibilities can significantly hinder the implementation of effective countermeasures. In contexts where health governance is decentralized, this often results in delays, inconsistent policies, and reduced efficiency in managing public health emergencies.

In addition to anthropogenic and ecological factors, climate change may also influence the epidemiology of ASF. Rising temperatures and altered precipitation patterns can affect vegetation cover, water availability, and the behavior and distribution of wild boar populations. The presence of green spaces, such as forested areas, peri-urban parks, and agricultural margins can serve as ecological corridors that facilitate movement and contact among susceptible hosts. These areas may also become more attractive to wildlife under changing climatic conditions, potentially increasing the risk of pathogen spillover and persistence. While further research is needed to quantify these effects, climate-related variables should be considered in future models and surveillance strategies addressing ASF and similar wildlife-borne diseases [40]. In this regard, geospatial technologies, including Geographic Information Systems (GISs), satellite remote sensing, and drone-based monitoring can provide valuable data for mapping land cover changes, detecting habitat fragmentation, and analyzing climatic trends. These tools enhance the spatial resolution and predictive power of epidemiological models, supporting more effective surveillance, early warning systems, and prophylactic interventions.

Despite the growing concern over African swine fever (ASF) outbreaks across Europe, there remains a notable gap in research that integrates remote sensing and GIS-based approaches to model the spatial distribution of the virus in relation to environmental and spatio-temporal variables. Most existing studies focus on epidemiological patterns or genetic characterization, often overlooking the role of landscape structure, habitat interfaces, and ecological corridors in shaping disease spread. In particular, few investigations have attempted to quantify how environmental features—such as land cover types, elevation gradients, and proximity to anthropogenic infrastructures—interact with temporal dynamics to influence the movement of infected wild boar populations. This study addresses that gap by leveraging high-resolution spatial data and geospatial modeling techniques to explore the influence of habitat interfaces on ASF diffusion in Northwestern Italy, offering a novel perspective that complements traditional epidemiological analyses.

Therefore, in this study, we adopt spatial analysis methods, with particular focus on GISs and remote sensing, to monitor and assess the spread of ASF across northwestern Italy (Piedmont and Liguria regions). The integration of spatial mapping with GISs and remote sensing has been widely recognized as an effective approach in epidemiology and environmental health studies, as it enables the identification of risk factors and the visualization of disease dynamics over large geographic areas [41,42,43,44,45,46,47,48]. These methods are advantageous because they allow the combination of environmental, demographic, and ecological data, thereby supporting a comprehensive One Health perspective for disease surveillance and control. The objective of this study is therefore to (i) identify high-risk areas by considering pig density, proximity to wildlife, and environmental conditions and (ii) characterize spatial and temporal patterns of ASF spread, in order to provide decision-makers with targeted prevention measures, including improved biosecurity protocols and more efficient surveillance strategies.

## 2. Materials and Methods

### 2.1. The Area of Interest

**Figure 1:**
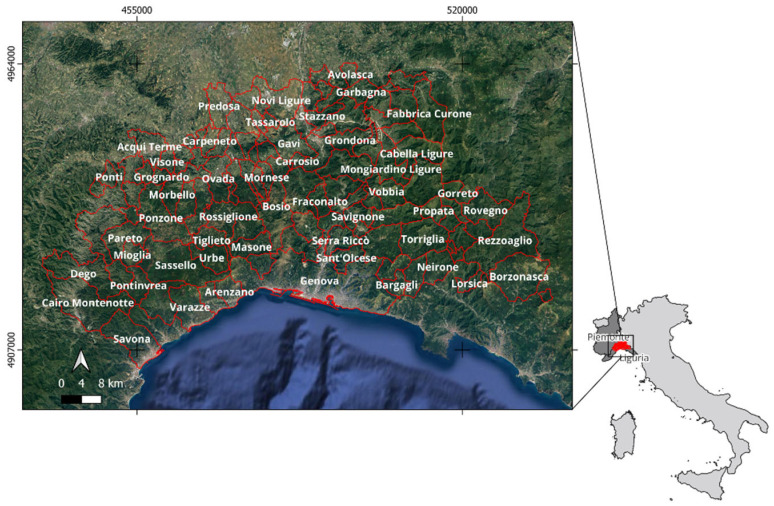
Study area involving Alessandria, Asti, Genoa, and Savona provinces in the Piemonte and Liguria regions (northwestern Italy) in the time range from 2022 to 2024.

The study focused on the area of interest (AOI) for ASF spread, from the point of origin to its spreading, from 11 February 2022, the date of the release of the first consistent epidemiological bulletin issued by the Experimental Zooprophylactic Institute of Piedmont, Liguria, and Aosta Valley (here-in-after called IZSPLV), following the identification of the first outbreak in the municipality of Ovada (Piedmont), recorded in Italy and confirmed by laboratory tests on 8 January 2022, up until 21 January 2024. The Italian regions affected in the northwest are Piedmont (P) and Liguria (L), particularly the municipalities shown in Figure 1, located in the provinces of Alessandria (P), Asti (P), Genoa (L), and Savona (L). Although pig farms are not densely distributed as in other regions of Italy, the spatial proximity to France and regions with numerous pig farms, such as Emilia Romagna and other provinces within Piedmont, such as the Cuneo province, make this area a subject of significant monitoring and attention.

### 2.2. Data Collection

#### 2.2.1. Epidemiological Data

Municipal boundaries in study area were collected in vector format with the highest level of detail from the Italian National Institute of Statistics (ISTAT) for the reference year 2024 (https://www.istat.it/notizia/confini-delle-unita-amministrative-a-fini-statistici-al-1-gennaio-2018-2/, last accessed on 20 January 2025). This layer has a nominal scale of 1:10,000 and was updated in 2024.

Meanwhile, ASF epidemiological data for the reference period from 11 February 2022 to 21 January 2024 were downloaded from the epidemiological bulletins available on the dedicated webpage provided by IZSPLV (https://www.izsplv.it/it/notizie/308-peste-suina-africana.html, last accessed on 20 January 2025). The data refers to the number of wild boars positive to ASF. Between January 2022 and January 2024, ASF outbreaks were confirmed in wild boar populations across several provinces in northwestern Italy. The first case was detected in Ovada (Alessandria Province, Piedmont) on 7 January 2022. Subsequent cases were reported in Liguria (Genoa and Savona provinces), Lazio (Rome and Rieti), Calabria (Reggio Calabria), Campania, and Lombardy (Pavia). The virus was detected exclusively in wild boars within the AOI, with no confirmed outbreaks in domestic pigs during the study period.

Since ASF bulletins were natively provided with geolocation at the municipality level, a table covering the reference period was created, and a spatial join with ISTAT municipality boundary data was performed to properly locate ASF data. It is worth noting that ASF epidemiological data were collected daily or with a delay of no more than three days, involving considerable team effort from personnel at IZSPLV and its peripheral offices, the Forestry Corps (Carabinieri Forestali), veterinarians within the ASL (Aziende Sanitarie Locali), citizen-reported findings, and support from hunters. In this regard, the Italian Ministries of Health and Agriculture and Food Sovereignty have mobilized financial resources for monitoring the epidemic, given the potential damage to the rural economy and the Italian agri-food sector, as demonstrated by public engagement on this issue (https://storymaps.arcgis.com/stories/9fe6aa3980ca438cb9c7e8d656358f35, last accessed on 20 January 2025).

#### 2.2.2. Land Use, Roads, and Trails Data

The land use/land cover (LULC) 2022 map, including major roads, was downloaded from the ISPRA (Italian Institute for Environmental Protection and Research) geoportal (https://groupware.sinanet.isprambiente.it/uso-copertura-e-consumo-di-suolo/library/copertura-del-suolo/carta-di-copertura-del-suolo/uso-del-suolo-2022, last accessed on 20 January 2025). This raster layer has a native spatial resolution of 10 m and 11 classes regarding the land use [49], specifically, forest use, quarries and mining, urban and assimilated areas, water uses, arable land, forage crops, permanent crops, agroforestry areas, other agricultural uses, wetlands, other non-economic uses [50,51,52,53]. The map was clipped in QGIS version 3.28.0 to the boundaries of the study area. Since the LULC map does not include secondary roads and trails, these were added by incorporating the liens vector layer of road network and trail layers from the geoportals of the Piemonte and Liguria regions, respectively, from (https://geoportale.igr.piemonte.it/cms/ and https://geoportal.regione.liguria.it/, last accessed on 20 January 2025) with a nominal scale of 1:2000 and updated in 2024. Since the road footprint was not always available, a 10 m buffer was applied to maintain consistency with the spatial resolution of the LULC raster. These products were used to map habitat interfaces and were considered in the spatio-temporal dynamics of the ASF spread and modeling.

#### 2.2.3. Livestock Data

**Figure 2:**
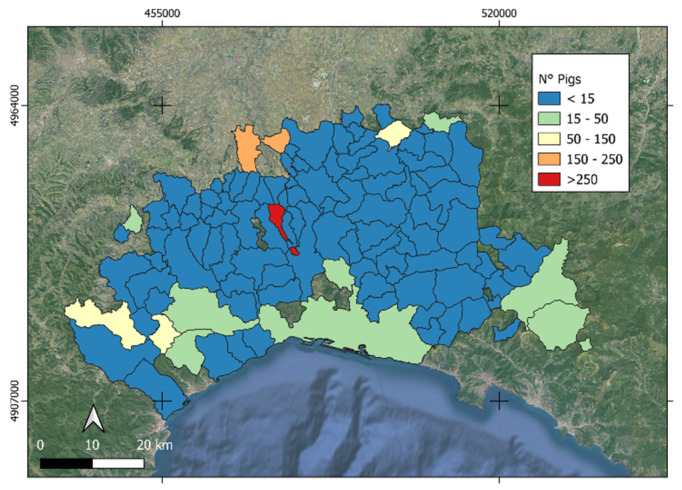
The number of pigs per municipality in the study area as of 21 January 2024. Municipalities with no pigs have not been reported.

Livestock data regarding pig populations and farm locations for the analyzed reference years were collected from ISTAT (https://esploradati.istat.it/databrowser/#/it/censimentoagricoltura, last accessed on 20 January 2025) and the BDN-Banca Dati Nazionale (https://www.vetinfo.it/j6_statistiche/#/, last accessed on 20 January 2025), see Figure 2. The Italian Veterinarian Database (BDN) requires all farms where pigs or wild boars are bred or kept to be registered, including family farms that hold even a single fattening animal for self-consumption [54]. This information is provided at municipality level and therefore was added to the municipalities layer by a table join procedure in QGIS.

### 2.3. Data Processing

**Figure 3:**
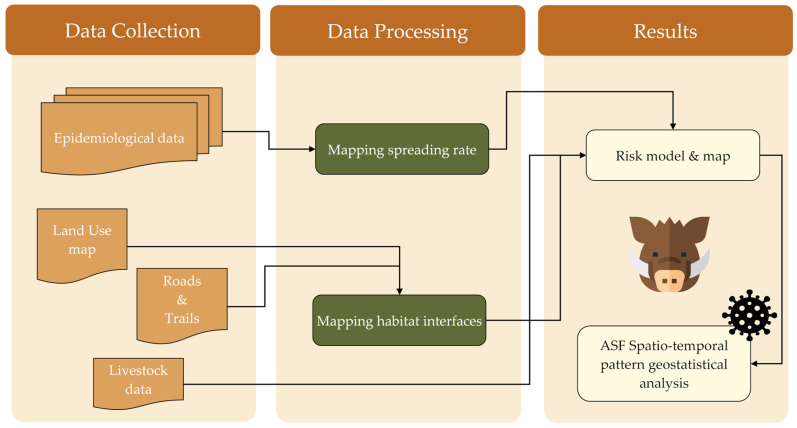
Adopted workflow for analyzing ASF spatio-temporal pattern.

The workflow adopted in this work is reported in Figure 3. Input data (ASF outbreaks, pig density, wildlife, environmental variables) were collected and processed through two main steps: mapping the spreading rate with a logistic model and mapping habitat interfaces. These outputs were integrated to develop a spatial risk model and corresponding risk map, followed by a geostatistical analysis. Further methodological details are provided in the following sections.

#### 2.3.1. Mapping the Spreading Rate

Using QGIS vs. 3.28 [55,56] epidemiological data tables were joined to the municipalities boundaries in order to spatially locate ASF cases. To synthesize such ASF disease temporal trends, for each municipality, a 3-parameter logistic model reported in Equation (1) was fitted using the “bmd” package in R vs. 4.1.1 [57,58].

(1) $$y\left(t\right)=\frac{d}{1+exp\left[b\left(t-e\right)\right]}$$

where *t* is time; *y* is number ASF-positive cases at *t*; d is the upper asymptote; *e* is the date of inflection point; and *b* is the growth rate of the disease (i.e., daily increase in positive cases).

Three maps of the corresponding model parameters were consequently created, namely the maximum number of cases in each municipality—*d(x,y)*; inflection point date—*e(x,y)*; and spreading rate—*b(x,y)*, where *x* and *y* denote cartographic coordinates and imply a dependence of values according to its position in space.

Also, the determination coefficient of regression was estimated and mapped into a new layer *R^2^(x,y)*. The latter was used to focus the analysis on trends showing a logistic-like temporal behavior. Since this condition may not be satisfied everywhere (i.e., some trends can be fitted by linear or high order polynomials), all trends showing a R^2^ lower than 50% were masked out so that the remaining ones ensured logistic assumption. Only municipalities showing a logistic-like temporal behavior (R^2^ ≥ 0.5) were retained for analysis, resulting in the exclusion of eight municipalities with poor fit. Although different temporal behaviors could in principle be modeled using alternative functions (e.g., linear, exponential, or higher-order polynomials), the logistic model was selected because it was well established in epidemiology, suitable for binary outcomes such as ASF presence or absence, and widely used to summarize temporal patterns of virus-driven epidemics [59,60,61]. Given the limited number of exclusions, we considered the logistic model sufficiently robust and generalizable for the purposes of this study.

Finally, since different starting conditions are expected between trends (i.e., higher number of positive cases at the beginning of the analyzed period) a normalized spreading rate (NSR) was computed in Equation (2)

(2) $$NRS=exp\left(b\right)/d$$

NSR allows for the proper comparison of spreading rates among the analyzed municipalities providing a spatial overview, *NSR(x,y)*, of the ASF outbreak.

To assess the uncertainty of this coefficient, we applied a nonparametric bootstrap approach, which does not require distributional assumptions. For each lag class, all point pairs separated by a distance within the class were identified and used to compute the empirical co-dispersion coefficient. To estimate confidence intervals, we performed bootstrap resampling: in each iteration, the same number of pairs as in the original lag class was randomly sampled with replacement. The co-dispersion coefficient was recalculated for each bootstrap sample, and this process was repeated 1000 times to generate an empirical distribution. The 95% confidence interval was then obtained from the 2.5th and 97.5th percentiles of the bootstrap distribution. This procedure accounts for the internal variability of each lag class and provides a robust measure of uncertainty, especially in the presence of spatial dependence and small sample sizes.

#### 2.3.2. Mapping the Habitat Interfaces

In the literature, the role of the local environment was deeply proved to affect animals-related disease spreading, especially influencing the infection [62].

Moreover, some recent research [57,58] highlighted the importance of wildlife habitat interface in fostering disease spreading. The majority of these interfaces are related to urban areas or roads/trails where humans and animals (i.e., vectors) can somehow interact.

Stimulated by this, in this work, to spatially analyze these factors, LULC and streets/roads maps were used. In particular, LULC urban and built-up areas were isolated and merged to the vector layer resulting in 10 m buffering [63] around streets/roads. Since wild boar habitats are strictly related to vegetated areas (forests or crops), forest and crop classes as classified from LULC maps were isolated and the Habitat Density—*HD(x,y)*—for each municipality was computed as the ratio between the habitat area and municipality area. Moreover, the interfaces between vegetated areas and streets/roads or urban areas were mapped as vector lines. The interface density (i.e., km ha^−1^) for each municipality was computed summing line lengths within municipality boundaries. This new layer called Interface Density—*ID(x,y)*—was created using this interpretative key: higher ID meant a higher the probability that humans could interact with ASF vectors (e.g., boars, soft ticks, or infected material) as previously suggested for different wildlife diseases in the literature [20,64,65].

#### 2.3.3. Livestock Risk Modeling

In order to assess how environmental factors can affect ASF spreading, a simple risk model [20] was proposed (Equation (3))

(3) $$R\left(x,y\right)=L\left(x,y\right)\cdot V\left(x,y\right)\cdot I\left(x,y\right)$$

where *R(x,y)* is the total risk, i.e., the probability that ASF negatively affects livestock pigs; *L(x,y)* is the likelihood, i.e., the probability to find infected vector; *V(x,y)* is the vulnerability, i.e., the probability to interact with a vector; and *I(x,y)* is the impact, i.e., the probability that a negative event affects targets (for ASF, the livestock animals). The risk and involved factors are explicitly spatial dependent as denoted by spatial coordinates *x* and *y*.

For all subsequent steps, all variables’ maps were normalized using the min-max normalization in order to express all data in a range [0;1] making possible a proper comparison between different measurements units.

*L(x,y)* was computed as the product between *HD(x,y)* and the average positive cases per municipality in the considered period. The former is the habitat density meaning that *L(x,y)* increases with higher vegetated areas because it is more probable to find wild boars/ticks. The latter somehow represents the *inoculum* present in a municipality; it takes care that positive cases may occur in a shorter or longer period, allowing a comparison between *L(x,y)* values. Therefore, their product can synthesize the probability of finding infected vectors in a given location. *V(x,y)* was identified as *ID(x,y)* since it represents the interface density. The latter describes the probability of interaction between vectors (humans and boars or natural infected materials).

It is worth highlighting that the product *L(x,y)* and *V(x,y)* here represent the environmental component of the risk—*ER(x,y)*. It means that local environmental conditions (i.e., inoculum, habitats, and urban extensions) are pivotal components of the ASF risk. Finally, *I(x,y)* quantifies the probability of damage on targets, computed as the livestock pig density per municipality. If no livestock pigs are present *I(x,y)* is 0, nulling the risk.

#### 2.3.4. Geostatistical Analysis of ASF Spatio-Temporal Pattern

To assess how local environmental conditions (here summarized in *ER(x,y)*) can affect spreading (summarized in *NSR(x,y)*), a multivariate geostatistical analysis was performed. In particular, the aim is to assess the structure of the spatial correlation of these variables, estimating the range within which a correlation may exist. Beyond this range is a reasonable assumption of a poor relationship between factors. To quantify the spatial pattern of such a relationship, the co-dispersion coefficient (Equation (4)) was computed from variograms and cross-variograms of *ER(x,y)* and *NSR(x,y)*. The assumption is that both ER and NSR are spatially varying, therefore also their spatial autocorrelation and cross-correlation have to vary. This type of analysis [66] can test for spatial relations between the two variables and can highlight the range (spatial distance) of validity of such relationships [66].

(4) $$\rho_{uv}\left(h\right)=\frac{\gamma_{uv}\left(h\right)}{\sqrt{\gamma_{uu}\left(h\right)\gamma_{vv}\left(h\right)}}$$

where $\rho_{uv}\left(h\right)$ is the co-disperison coefficent between the *u-th* and *v-th* variables at lag distance **h**; increasing $\rho_{uv}$ value means a higher correlation. $\gamma_{uu,vv}\left(h\right)$ is the auto-variogram of the *u-th* or *v-th* variable computed as in Equation (5), while $\gamma_{uv}\left(h\right)$ is the value obtained by a cross-variogram computed in Equation (6).

(5) $$\gamma \left(h\right)=\frac{1}{2m\left(h\right)}\sum_{i=1}^{m\left(h\right)}{\left[z\left(x_{i}\right)-z\left(x_{i}+h\right)\right]}^{2}$$

where $z\left(x_{i}\right)$ is the value of the spatial variable at the $x_{i}$**-***th* location, while $z\left(x_{i}+h\right)$ is the value at the location lagged of h distance.

(6) $$\gamma_{uv}\left(h\right)=\frac{1}{2m\left(h\right)}\sum_{i=1}^{m\left(h\right)}\left\{\left[z_{u}\left(x_{i}\right)-z_{u}\left(x_{i}+h\right)\right]\left[z_{v}\left(x_{i}\right)-z_{v}\left(x_{i}+h\right)\right]\right\}$$

where $\gamma_{uv}\left(h\right)$ is the cross-variance between *u-th* and *v-th* at lag h.

Finally, to evaluate the proportion of variance as reported in Section 2.3.1, explained by the model, we used a variance-based determination coefficient index. This index was computed as

(7) $$R^{2}=1-\frac{var\left(residuals\right)}{var\left(observations\right)}$$

where var(residuals) is the variance of model residuals and var(observations) is the variance of the observed response variable. This measure reflects the relative reduction in variance achieved by the model compared with a null model and has been used in similar contexts for generalized or nonlinear models [67,68].

## 3. Results

### 3.1. Mapping the Spreading Rate

**Figure 4:**
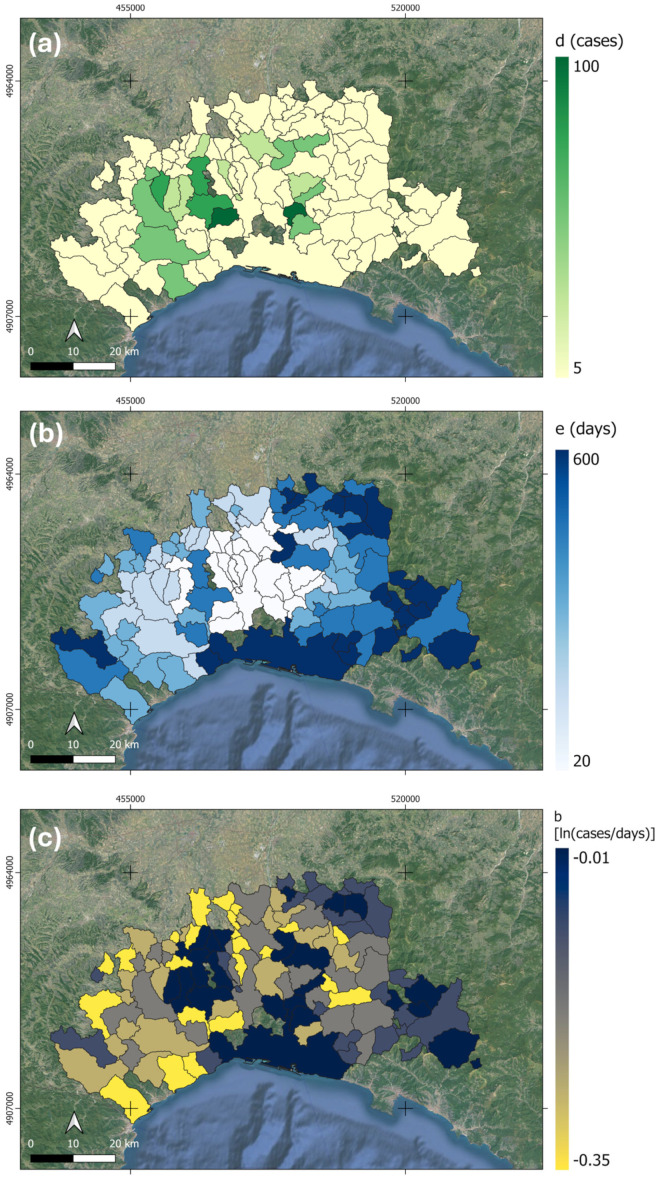
The 3-parameter logistic model coefficients were mapped, respectively, in figure (**a**) spatial distribution of upper asymptote (reported as *d*, maximum number of positive cases recorded in a given municipality), (**b**) the *e* parameter map (representing the date of implication point), and (**c**) the spreading rate (reported as *b* value, the increase in cases per days).

The three-parameter logistic model coefficients were mapped as shows in Figure 4. Moreover, eight municipalities were removed from the analysis since they showed a R^2^ value lower than 0.5 denoting a poor fitting of the logistic model. In Figure 4a, the spatial distribution of upper asymptote (maximum number of positive cases recorded in a given municipality) is shown. It can be noted that the majority of municipalities (about 60%) had *d* values lower than 10 cases with a clear pattern located in the center of AOI, denoting a local hotspot. In Figure 4b, the *e* parameter map (i.e., the date of inflection point) is shown. It can be interpretated as the temporal expansion of ASF. In fact, it can be noted that in the AOI center, the ASF trends started 20 days after 11 February 2022 while the municipalities in the most center part had a delayed trend (about 500 days after). Finally, in Figure 4c, the *b* value is reported. It represents the spreading rate, i.e., the increase in cases per day.

**Figure 5:**
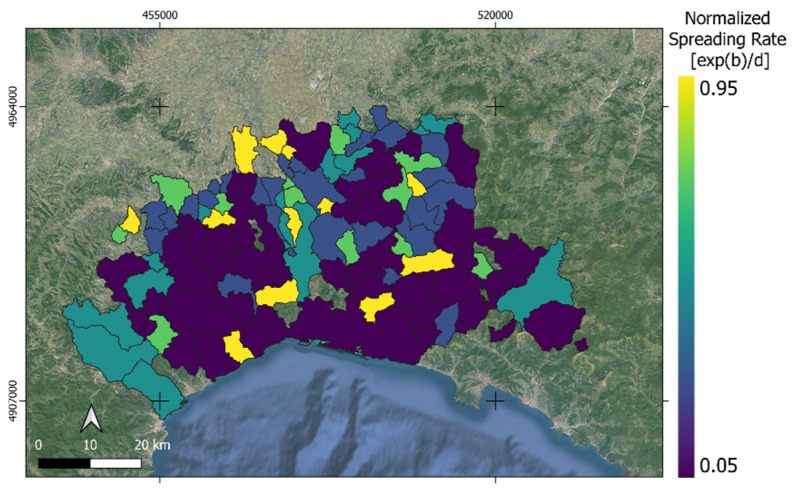
The spreading rate normalized by the upper asymptote, summarizing both the contagion effect and the speed of ASF.

Since the same spreading rate can denote different magnitudes of inoculum, the NSR was computed (Figure 5). The latter represents the spreading rate normalized by the upper asymptote; it somehow summarizes both the contagion effect and the speed of the ASF. The co-dispersion analysis revealed that the association between the two spatial variables is strongest at short distances and gradually decreases with increasing spatial lag. This pattern suggests that the processes influencing ASF dynamics in wild boar populations exhibit strong local dependence, likely driven by localized transmission mechanisms and environmental or landscape connectivity.

At small lags, the co-dispersion index is positive and statistically significant, as indicated by the 95% confidence intervals that do not overlap zero. This confirms that the two variables exhibit a consistent spatial association at local scales, likely driven by localized transmission processes and environmental connectivity. As the spatial lag increases, the co-dispersion index declines and its confidence intervals widen, approaching zero after a 60 km lag. This indicates increasing uncertainty and the absence of a significant spatial relationship at broader scales, consistent with the limited dispersal capacity of wild boars and the focal nature of ASF spread. The observed decay and loss of significance at larger distances highlight that spatial dependence is primarily a local phenomenon, which supports the importance of localized control measures in managing ASF risk. The observed decay in co-dispersion aligns with previous findings in spatial epidemiology, where pathogen transmission often shows strong spatial clustering at small scales, reflecting both biological and anthropogenic drivers. This highlights the importance of implementing control measures that account for local connectivity, particularly within the range where co-dispersion remains significant.

### 3.2. Mapping the Habitat Interfaces

**Figure 6:**
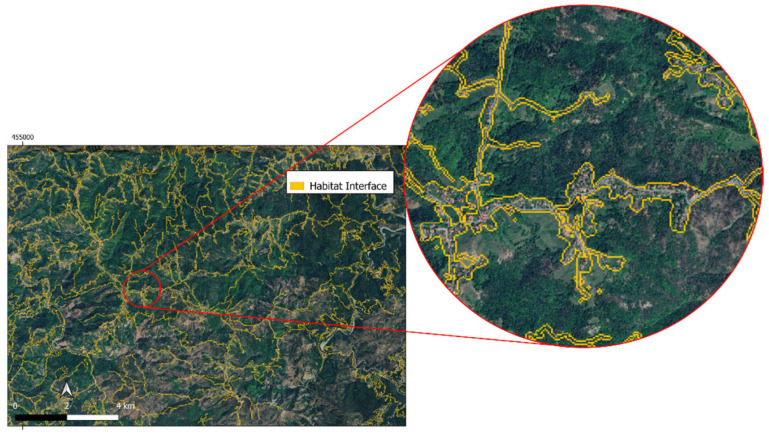
A spatial overview of AOI part and a zoom-in of habitats interfaces (yellow).

Figure 6 shows the habitats interfaces computed by LULC and streets/trials map intersections.

**Figure 7:**
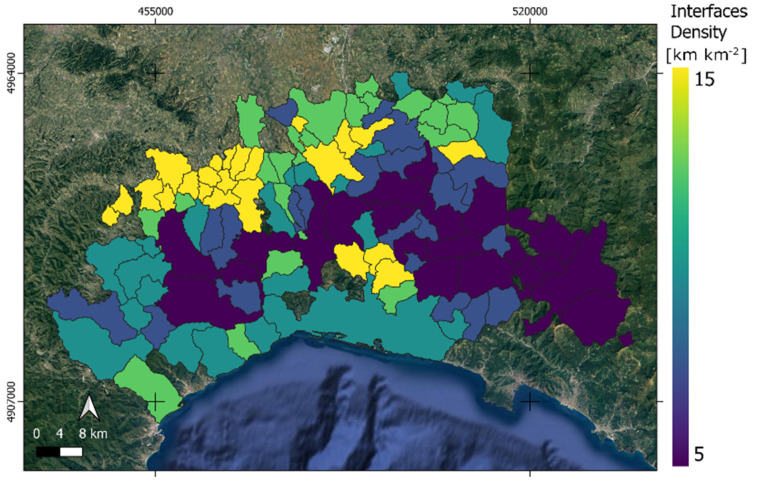
The Habitat Interface ID (x,y) density distribution within the AOI. Yellow colors denote high density in terms of lengths per areas within each municipality.

By comparing the lengths of interfaces by municipality area, the *ID(x,y)* layer was computed in order to spatially compare different municipalities (Figure 7). It can be noted that, in general, higher ID values are close to the north part of AOI, while lower values are present in the most center part. An interesting small concentration of high ID value is placed in the center of the AOI.

### 3.3. Livestock Risk Modeling

**Figure 8:**
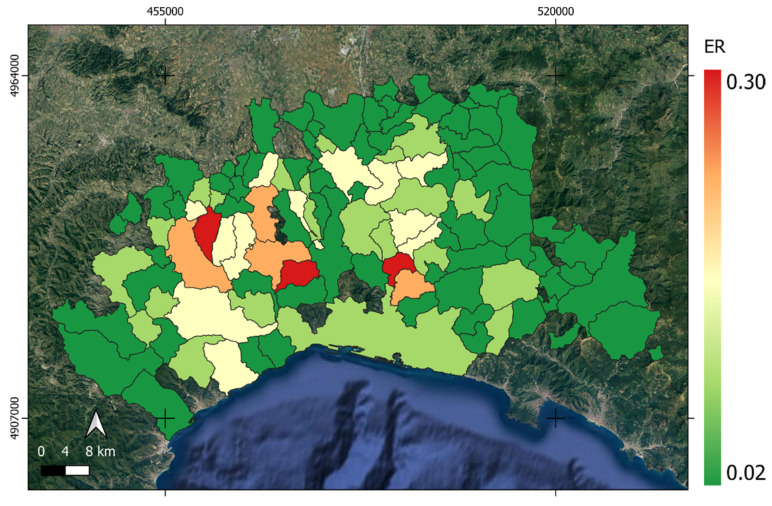
ER(x,y) is the environmental risk map representing the risk component independent of livestock presence. This component is mainly influenced by interface density (ID) and the inoculum level within each municipality. Higher ER values are concentrated in the central–western part of the AOI (yellow to red areas), while risk gradually decreases toward the boundaries (light to dark green areas).

Assuming the risk model proposed in Equation (3), Figure 8 shows *ER(x,y)*. The latter is the environmental component of the risk, and it is independent from livestock presence. It is mainly related to ID and the inoculum present in a given municipality. This risk component is higher in the center-west of the AOI while tending to reduce at the AOI limits.

**Figure 9:**
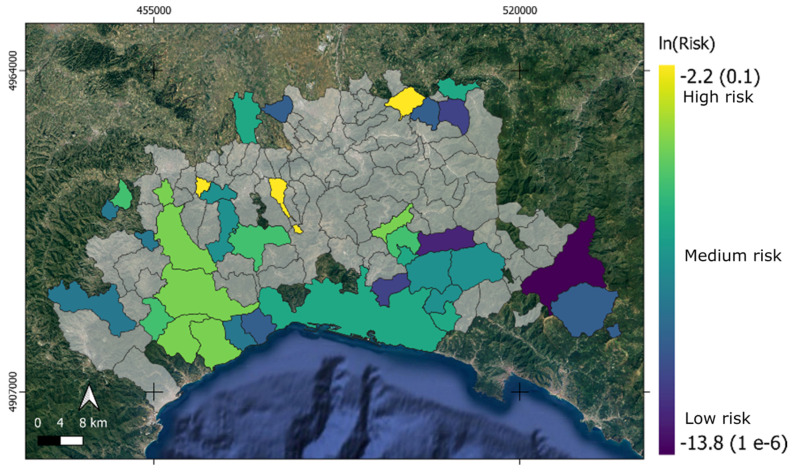
Map of the total ASF risk. Most municipalities show minimal risk (gray) due to low I(x,y) values and limited pig presence. Higher risks are observed in the central–western areas, while southern and eastern regions exhibit lower risk levels.

Figure 9 shows the total ASF risk. It is apparent from this map that most municipalities have a risk close to 0. This is not unexpected, since the *I(x,y)* value is low, as also supported by Figure 2 (ISTAT data), where few or zero pigs are present. This null total risk is due to the lack of ASF target hosts.

However, in the remaining municipalities, a higher risk is observed, particularly in the central–western part of the AOI, with a peak risk in the Garbagna municipality (north). Currently, the southern and eastern parts show lower risk values.

### 3.4. Geostatistical Analysis of Spreading Spatio-Temporal Pattern

**Figure 10:**
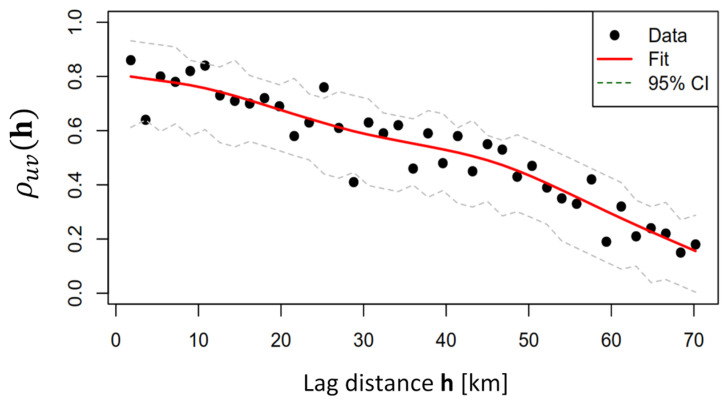
Co-dispersion index at **h** lag between ER (environmental risk) and NSR (spreading). The dispersion coefficient shows a positive correlation with differing spatial patterns based on spatial lag. Higher correlations (>0.6) occur at distances under 20 km, while the correlation weakens beyond 50 km, indicating that ER has no significant impact on spreading rates at larger distances.

Although low-risk values were, in general, found in the AOI in the considered period, unexpected spreading behaviors (like the ones generated by human-related jump dispersion) can lead in future due to a more complicated ASF control. To create an ASF control management tool for future scenarios and explore how local environment conditions can foster spreading, a geostatistical analysis was performed. In Figure 10, the co-dispersion coefficient between NSR and ER is shown. It can be noted that they are positively correlated, but their spatial patterns co-vary differently according to spatial lag. In fact, higher correlation (>0.6) is found at lag distances lower than 20 km, while for higher distance correlation is lower. This phenomenon denotes that cross-correlation decreases with municipality distances suggesting that after 50 km (the elbow of the cross-correlogram) significantly approaches zero. After this range, ER has no significant effects on spreading rates.

## 4. Discussion

The model developed in this study offers significant insights into the spatio-temporal dynamics of ASF spread within the area of interest, with important policy implications for the control and management of the disease in the regions of Piedmont and Liguria. The observed strong spatial association between environmental risk (ER) and ASF spread (NSR) at short spatial lags, particularly below 20 km, suggests that local environmental conditions play a critical role in facilitating transmission. Consequently, disease control strategies should prioritize fine-scale interventions targeting high-risk zones identified through environmental risk assessments. Localized measures, such as the deployment of barriers, increased surveillance, and carcass removal, are likely to be more effective in disrupting transmission pathways where ER is most strongly correlated with spreading dynamics. Beyond the 20 km threshold, however, the weakening of the co-dispersion index, and its near absence at distances greater than 50 km, indicates that ER loses explanatory power for disease propagation at broader spatial scales. This highlights that long-distance ASF transmission may instead be driven by factors such as human-mediated movements, hunting practices, or accidental translocations. Therefore, at larger scales, policy efforts should shift from environmentally driven interventions toward broader biosecurity measures and inter-regional coordination to monitor and mitigate these alternative vectors of spread.

Overall, these results support a scale-sensitive approach to ASF management, in which interventions are tailored to the relevance of environmental risk at different distances. High-resolution risk mapping and a concentration of resources in areas of strong ER–NSR correlation can improve control efficiency, avoid dispersion of resources in low-risk areas, and foster sustainable long-term management.

Like any model, ours has limitations. The exclusion of eight municipalities due to poor logistic fit (R^2^ < 0.5) may bias results locally, but the three-parameter logistic model was retained as the most general option, consistent with other virus-driven epidemics [33,69]. Reporting delays and under-reporting of ASF-positive carcasses also remain critical issues. Our use of parameters such as the inflection point (e), growth rate (b), and asymptote (d), as well as the normalization index b/d, reduces—but does not eliminate—the bias introduced by incomplete data. Future work should include sensitivity analyses or correction methods to account for these gaps.

Another limitation is the inability to fully capture human-related “jump dispersal” events, such as the movement of infected pigs or indirect human-mediated transmission, which can alter epidemic dynamics unpredictably [70]. Integrating human behavior factors, such as foot traffic data from GNSS/SDK providers (e.g., Echo Analytics, Lifesight, Gravy Analytics, last accessed 6 February 2025), could substantially improve predictive robustness.

The logistic model was chosen for its suitability for binary outcomes, capacity to estimate probabilities based on exogenous variables, and flexibility in incorporating predictors such as habitat interfaces and remotely sensed environmental data. This framework has highlighted significant regional variability—for example, higher asymptote (d) values marking hotspots in the AOI center, contrasted with delayed ASF dynamics in the east. Although assumptions of constant animal density and inoculum levels oversimplify reality, the model still enables spatially explicit prevention planning, which is highly relevant given the economic importance of pig farming in Italy.

One strength of this work is its integration of environmental data into risk assessment. By combining geospatial and geostatistical approaches [71,72,73], the model demonstrated that local environmental conditions strongly influence ASF spread, underlining the need to identify regional hotspots. Although post hoc validation with farm outbreak data was not possible—due to successful mitigation efforts and lack of georeferencing—future studies should leverage new spatial data for model validation and refinement.

Spatial–temporal modeling and geostatistical approaches have been widely employed in the study of various animal and zoonotic diseases beyond ASF. For instance, similar methodologies have been used to monitor the spread of bovine tuberculosis in wild boar and red deer populations in Portugal [74], to assess the spatial dynamics of rabies using GIS-based real-time mapping tools [53] and model the habitat suitability and landscape connectivity for tick-borne diseases such as Lyme disease [75]. Moreover, the integration of habitat interfaces and environmental variables has proven effective in predicting the distribution of Bartonella spp. in fox populations [46,76] and in mapping the risk of canine distemper virus in wildlife using NDVI-based remote sensing techniques [29]. These examples underscore the versatility and relevance of spatial epidemiology and geostatistics in understanding disease ecology, guiding surveillance efforts, and informing targeted interventions across a range of infectious diseases.

The use GIS and remote sensing provides additional opportunities for ASF risk modeling [12,28,57]. These tools allow the mapping of ER and habitat interface density (ID) and can capture temporal changes in vegetation, land cover, and habitat conditions changes [75,77,78,79,80,81,82,83,84,85]. Since wild boar distribution often correlates with habitat type, such data can serve as valuable proxies where population estimates are unavailable. Multispectral satellite imagery can further support dynamic models that incorporate seasonal, land use, or climate-driven changes, helping predict ASF transmission more accurately [12]. Incorporating meteorological variables, such as temperature, humidity, and precipitation, would also enhance predictive accuracy, given their influence on virus persistence and host mobility [86,87].

Future work should also focus on anthropogenic drivers of ASF spread. Datasets from analytics providers and national veterinary platforms (SIV, SIMAN, last accessed 6 February 2025) could be integrated with GISs [76,88,89,90,91,92,93,94,95] and socio-economic data on livestock practices. This would enable models that capture both environmental and human-related factors [64,96,97,98], reflecting empirical evidence of their role in ASF transmission [74,99,100,101,102,103,104,105,106,107,108,109,110,111].

Finally, increasing spatial resolution with georeferenced data and incorporating additional risk factors, such as climate and wildlife densities, would further enhance the model’s precision and adaptability across regions. These improvements would support more context-specific control strategies, ultimately reducing both the economic and ecological impacts of ASF outbreaks.

## 5. Conclusions

This study demonstrates the value of spatial analysis, particularly a GIS-based one, in monitoring and modeling the spread of ASF in northwestern Italy. By integrating spatial and temporal information, the analysis highlights the importance of habitat interfaces and local environmental conditions in shaping disease transmission dynamics. These findings have clear practical implications, as they provide decision-makers with tools to identify high-risk areas and design targeted prevention strategies, including surveillance, biosecurity measures, and rapid response interventions.

From a theoretical perspective, the study contributes to the growing body of research demonstrating how spatial modeling can improve our understanding of complex disease systems, particularly those involving wildlife–livestock interactions. However, the predictive capacity of the proposed framework is limited by data availability and resolution, as well as by factors not fully captured in the model, such as human-mediated movement and hunting practices. These limitations highlight the need for caution in extrapolating results and suggest that complementary approaches are necessary to address ASF spread at broader spatial scales.

Future research should therefore focus on integrating additional ecological and socio-economic datasets, refining modeling approaches and testing the applicability of the method in different geographical and epidemiological contexts. Such developments would enhance the predictive power of spatial models and strengthen their role as decision-support tools in animal health management.

## Data Availability

Data can be accessed at https://www.izsplv.it/it/notizie/308-peste-suina-africana.html (accessed on 8 August 2025).
